# Supplementary material for: Resolving the full spectrum of human genome variation using Linked-Reads
Source: Genome Res. 2019 Apr;29(4):635–45. doi: 10.1101/gr.234443.118 (PMC6442396; doi:10.1101/gr.234443.118)
Supplement: Supplemental Material [file supp_gr.234443.118_Supplemental_longranger-2.2.zip › longranger-2.2.tar.bz2!/lr2.2-public/lib/stan/cmdstan/stan/doc/api/html/namespacestan_1_1json.html]

Stan: stan::json Namespace Reference


|  |  |
| --- | --- |
|  | Stan  2.5.0  probability, sampling & optimization |


- Main Page
- Related Pages
- Namespaces
- Classes
- Files

- Namespace List
- Namespace Members

All Classes Namespaces Files Functions Variables Typedefs Enumerations Enumerator Friends Macros Pages

- stan
- json

Classes |
Functions

stan::json Namespace Reference

|  |  |
| --- | --- |
| Classes | |
| class | json\_data |
|  | A `json_data` is a `var_context` object that represents a set of named values which are typed to either `double` or `int` and can be either scalar value or a non-empty array of values of any dimensionality. More... |
|  | |
| class | json\_data\_handler |
|  | A `json_data_handler` is an implementation of a `json_handler` that restricts the allowed JSON text a set of Stan variable declarations in JSON format. More... |
|  | |
| struct | json\_error |
|  | Exception type for JSON errors. More... |
|  | |
| class | json\_handler |
|  | Abstract base class for JSON handlers. More... |
|  | |

|  |  |
| --- | --- |
| Functions | |
| template<bool Validate\_UTF\_8, typename Handler > | |
| void | parse (std::istream &in, Handler &handler) |
|  | Parse the JSON text represented by the specified input stream, sending events to the specified handler, and optionally validating the UTF-8 encoding. More... |
|  | |
| template<typename Handler > | |
| void | parse (std::istream &in, Handler &handler) |
|  | Parse the JSON text represented by the specified input stream, sending events to the specified handler, and optionally validating the UTF-8 encoding. More... |
|  | |

## Function Documentation

template<bool Validate\_UTF\_8, typename Handler >

|  |  |  |  |
| --- | --- | --- | --- |
| void stan::json::parse | ( | std::istream & | *in*, |
|  |  | Handler & | *handler* |
|  | ) |  |  |

Parse the JSON text represented by the specified input stream, sending events to the specified handler, and optionally validating the UTF-8 encoding.

Template Parameters
:   |  |  |
    | --- | --- |
    | Validate\_UTF\_8 |  |
    | Handler |  |

Parameters
:   |  |  |
    | --- | --- |
    | in | Input stream from which to parse |
    | Handler | Handler for events from parser |

Definition at line 429 of file json\_parser.hpp.

template<typename Handler >

|  |  |  |  |
| --- | --- | --- | --- |
| void stan::json::parse | ( | std::istream & | *in*, |
|  |  | Handler & | *handler* |
|  | ) |  |  |

Parse the JSON text represented by the specified input stream, sending events to the specified handler, and optionally validating the UTF-8 encoding.

Template Parameters
:   |  |  |
    | --- | --- |
    | Validate\_UTF\_8 |  |
    | Handler |  |

Parameters
:   |  |  |
    | --- | --- |
    | in | Input stream from which to parse |
    | Handler | Handler for events from parser |

Definition at line 445 of file json\_parser.hpp.


---

[ Stan Home Page ]

*© 2011–2014,
Stan Development Team.*
